# Evaluation of three read-depth based CNV detection tools using whole-exome sequencing data

**DOI:** 10.1186/s13039-017-0333-5

**Published:** 2017-08-23

**Authors:** Ruen Yao, Cheng Zhang, Tingting Yu, Niu Li, Xuyun Hu, Xiumin Wang, Jian Wang, Yiping Shen

**Affiliations:** 1 Department of Medical Genetics and Molecular Diagnostic Laboratory, Shanghai Children’s Medical Center, Shanghai Jiaotong University School of Medicine, Shanghai, 200127 China; 2 Boston Children’s Hospital, Boston, MA 02115 USA; 3 Department of Endocrinology and Metabolism, Shanghai Children’s Medical Center, Shanghai Jiaotong University School of Medicine, Shanghai, 200127 China

**Keywords:** Clinical sequencing, Copy number variants, Whole exome sequencing, Structural variation

## Abstract

**Background:**

Whole exome sequencing (WES) has been widely accepted as a robust and cost-effective approach for clinical genetic testing of small sequence variants. Detection of copy number variants (CNV) within WES data have become possible through the development of various algorithms and software programs that utilize read-depth as the main information. The aim of this study was to evaluate three commonly used, WES read-depth based CNV detection programs using high-resolution chromosomal microarray analysis (CMA) as a standard.

**Methods:**

Paired CMA and WES data were acquired for 45 samples. A total of 219 CNVs (size ranged from 2.3 kb – 35 mb) identified on three CMA platforms (Affymetrix, Agilent and Illumina) were used as standards. CNVs were called from WES data using XHMM, CoNIFER, and CNVnator with modified settings.

**Results:**

All three software packages detected an elevated proportion of small variants (< 20 kb) compared to CMA. XHMM and CoNIFER had poor detection sensitivity (22.2 and 14.6%), which correlated with the number of capturing probes involved. CNVnator detected most variants and had better sensitivity (87.7%); however, suffered from an overwhelming detection of small CNVs below 20 kb, which required further confirmation. Size estimation of variants was exaggerated by CNVnator and understated by XHMM and CoNIFER.

**Conclusion:**

Low concordances of CNV, detected by three different read-depth based programs, indicate the immature status of WES-based CNV detection. Low sensitivity and uncertain specificity of WES-based CNV detection in comparison with CMA based CNV detection suggests that CMA will continue to play an important role in detecting clinical grade CNV in the NGS era, which is largely based on WES.

**Electronic supplementary material:**

The online version of this article (doi:10.1186/s13039-017-0333-5) contains supplementary material, which is available to authorized users.

## Background

Copy number variants are important human genomic variants known to be responsible for Mendelian disorders as well as for common genetic conditions such as autism, intellectual disability, and schizophrenia [1–3]. Chromosomal microarray analysis (CMA) has demonstrated its technical validity and has remained the method of choice for the detection of genome-wide copy number variants (CNVs) in clinical settings. It has also demonstrated its clinical validity for both pre- and postnatal diagnostic testing [4, 5]. CMA is currently regarded as the gold standard for detection of CNVs that range from several kilobases to several megabases in size [6, 7].

The advent of next-generation sequencing (NGS) technology has dramatically improved our capability for examining small-scale sequence variants; it has also provided new options for the evaluation of large scale structural variants such as CNVs [8]. Whole-exome sequencing (WES) has been accepted as the most comprehensive test currently implemented in the clinical setting for small sequence variants [9, 10]. Much effort has been focused to generate CNV information from WES data [11]; however, low sensitivity and high false positive rates have been reported in previous studies using cancer cell lines [12], publicly available exome data [13], or comparing with whole genome sequencing data based CNV calling [14–16]. Thus, its technical validity has yet to be thoroughly evaluated.

Here, we evaluated three representative and popular read-depth based CNV detection programs: the eXome-Hidden Markov Model (XHMM), the Copy Number Inference From Exome Reads (CoNIFER), and CNVnator using clinical grade WES data. XHMM and CoNIFER detect rare CNVs based on a batched-comparison principle, while CNVnator detects CNVs based on a mean-shift approach within single samples. CNVs detected from the CMA platform were used as reference standard.

## Methods

### Samples and ethics statement

A total of 45 clinical diagnostic samples were enrolled from the Shanghai Children’s Medical Centre and the Maternal and Child Health Hospital of the Guangxi Zhuang autonomous region with the approval of respective institutional ethics review committees. Genomic DNA was extracted using the QIAamp Blood DNA Mini kit® (Qiagen GMBH, Hilden, Germany).

### WES and WES-based CNV detection

Exome targets were captured using the Agilent SureSelect Human All Exon V4 or V5 kit (Agilent Technologies, Santa Clara, CA). Raw sequencing data (FASTQ format) were generated via the Illumina HiSeq 2000 platform (Illumina, Inc., San Diego, CA). The Burrows Wheeler Alignment tool (BWA) v0.2.10 [17] was employed for sequencing data alignment to the Human Reference Genome (NCBI build 37, hg 19). All data were assessed using FastQC (version 0.11.2) (http://www.bioinformatics.babraham.ac.uk/projects/fastqc/) for quality.

CNVs were generated using the following three CNV detection programs: (1) XHMM v1.0 [18], (2) CoNIFER v0.2.2 [19], and (3) CNVnator v0.2.7 [20]. XHMM includes several analytic steps and involves a number of parameters. In our study, we set all parameters to default (minTargetSize: 10; maxTargetSize: 10,000; minMeanTargetRD: 10; maxMeanTargetRD: 500; minMeanSampleRD: 25; maxMeanSampleRD: 20; maxSdSampleRD: 150) for filtering samples and targets, and prepared the data for normalization via XHMM. The only parameter that could be adjusted on Conifer was SVD, which was set to 1. For CNVnator, we set the bin size to 50–60 according to the average coverage depth of our sequencing data (45–70 X). XHMM and CoNIFER used a pooled sample calling approach as input, and CNVnator called CNVs sample by sample after individually generating a baseline.

### CMA and CMA-based CNV detection

CMA were performed using three different array platforms including the SurePrint G3 customized array (Agilent Technologies, Santa Clara, CA), CytoScan HD (Affymetrix, Santa Clara, CA), and Infinium iSelect HD and HTS Custom Genotyping BeadChips (Illumina, San Diego, CA). Prior validated settings for each platform were consistently utilized for CNV detection and filtering. CNVs in the size range of 2 kb – 400 kb were detected via CMA and were further confirmed by manual inspection.

## Results

### Quality control of WES data

Fourteen samples were prepared using the Agilent SureSelect Human All Exon V4 kit and the remaining samples were prepared using the V5 kit. The mean read depth of all samples ranged around 50 X and the average read quality was well above the standard of 20 X. Details of sequence data are available in the supplemental data (Additional file 1: Table S1).

### Size distribution of CNV detected via CMA and WES

A total of 219 CNVs were detected via CMA from all samples. Forty-eight CNVs were located in regions that had no exome capture probes; consequently, they were removed from being used as true CNVs when comparing data between CMA and NGS. The remaining 171 CNVs were in regions involving at least one exon. The CNVs were examined and compared for size distribution, detection sensitivity, boundaries, and overlap among three programs and between two platforms.

1. CNV size

**Fig. 1:**
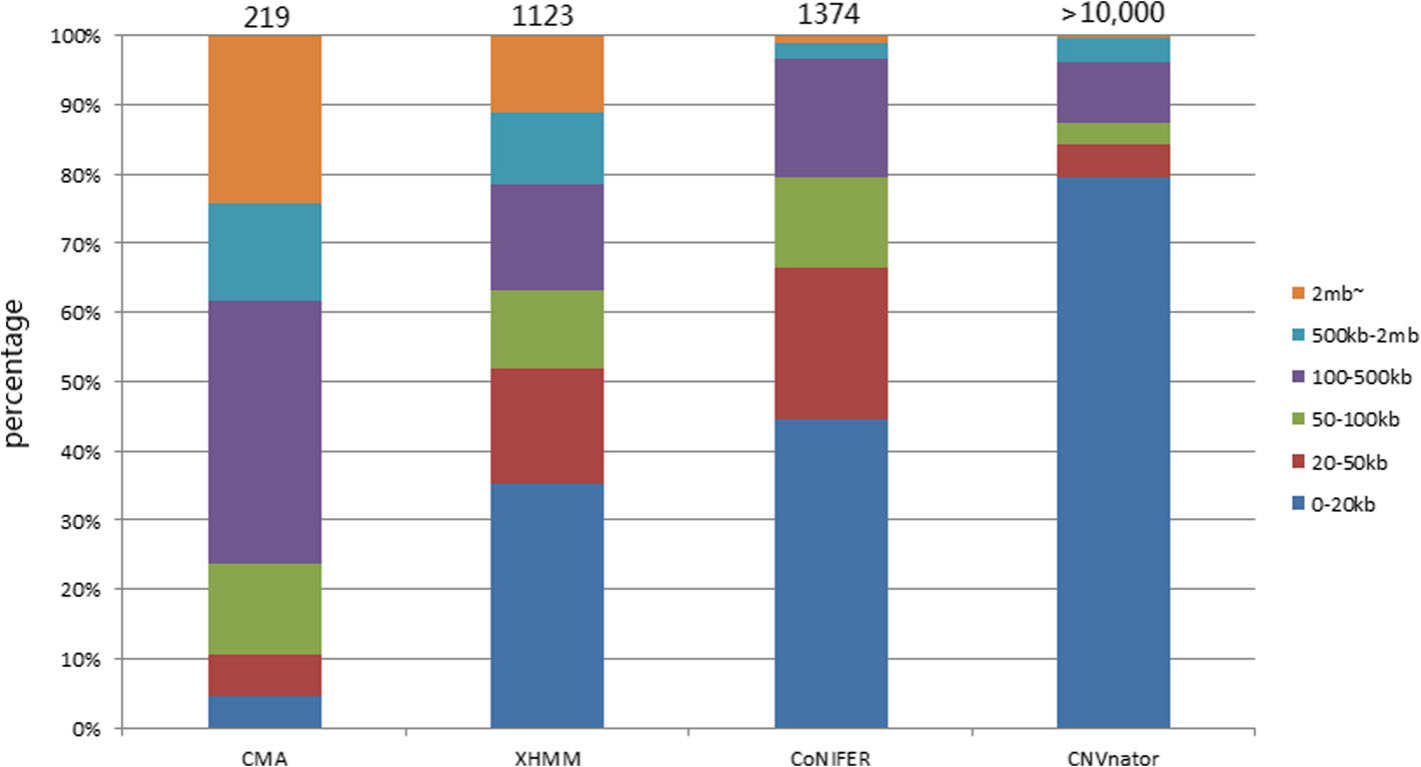
Number and size distribution of CNVs detected by CMA, XHMM, CoNIFER, and CNVnator

We arbitrarily constructed six size bins as shown in Fig. 1. The largest portion (37.9%) of CNV detected by CMA ranged within 100–500 kb whereas CNV detected by NGS data were of much smaller size; 35.3, 44.5 and 79.5% of CNVs were detected by XHMM, CoNIFER, and CNVnator, respectively and belong to the 0–20 kb bin. CNVnator in particular detected many smaller CNVs (42.2% below 10 kb, 27.3% below 5 kb).

2. Detection sensitivity

**Fig. 2:**
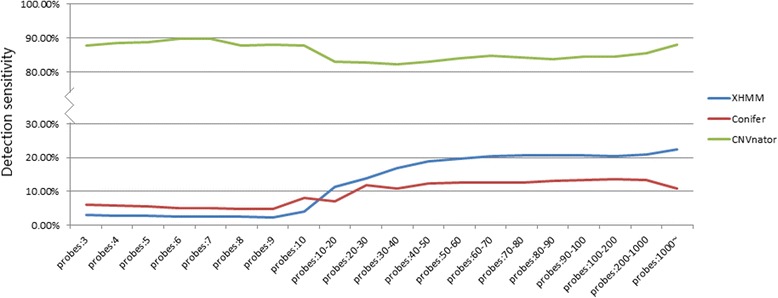
Sensitivity of three tools with different probe numbers within variants

We defined the detection of any particular CNV when there was a 50% overlap with a CNV detected via CMA. Using this definition for the presence/absence of CNV, 25, 38, and 150 CNVs were found to be detected by CoNIFER, XHMM, and CNVnator respectively; thus, the detection sensitivities of three programs were 14.6, 22.2 and 87.7%, respectively. CoNIFER and XHMM have an even poorer detection sensitivity for smaller CNVs involving fewer capturing probes, whereas CNVnator had a rather consistent detection sensitivity for all size CNVs (> 3 kb) (Fig. 2).

3. Precision of CNV detection

**Fig. 3:**
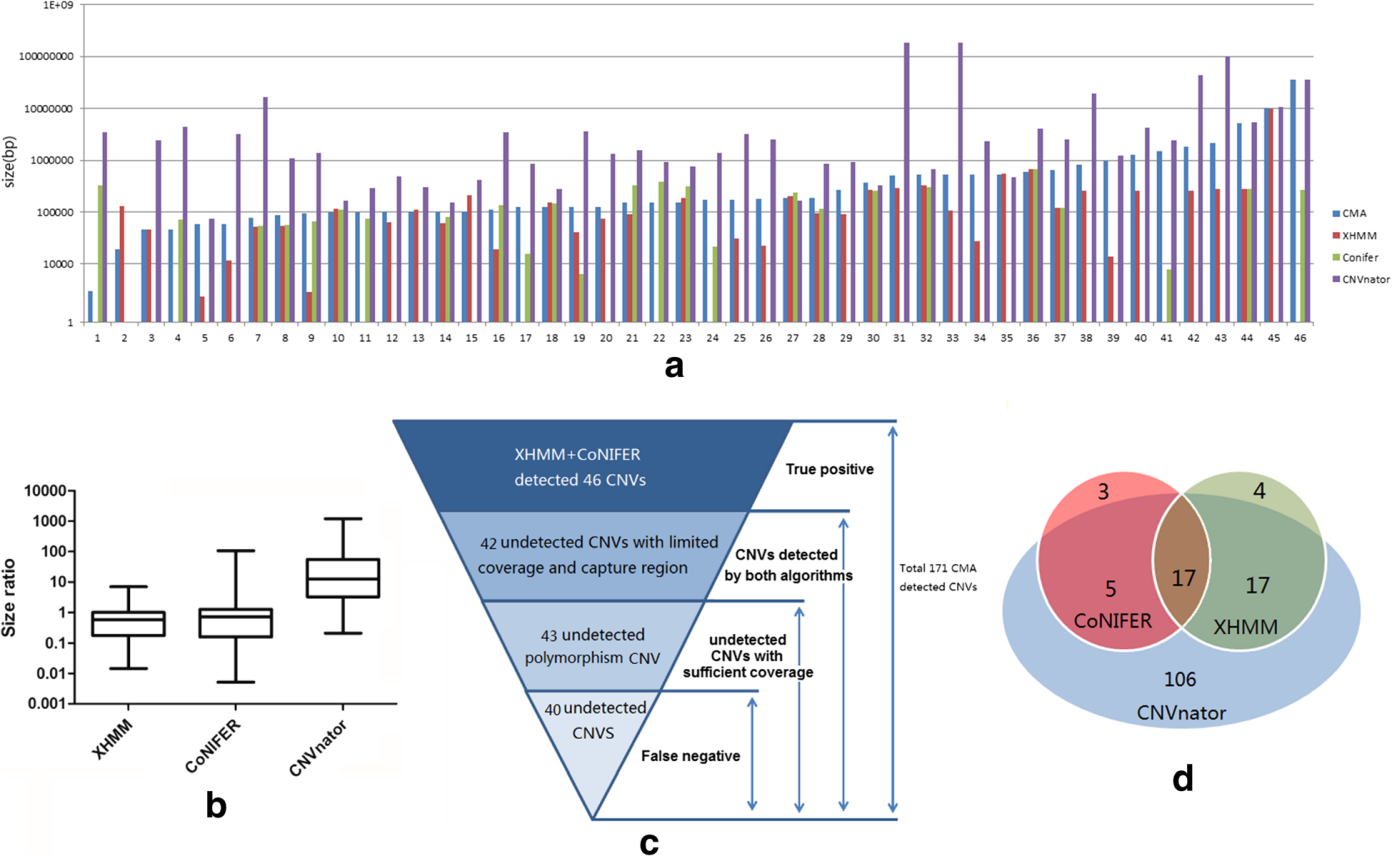
Evaluation of three CNV detection programs (XHMM, CoNIFER, and CNVnator) using clinical grade paired WES and CMA datasets. (**a**) 46 Variants detected by at least two different algorithms are listed ordered by size. (**b**) Size detected by three different programs in comparison with size detected by CMA. (**c**) Analysis of CNVs undetected by XHMM and CoNIFER. (**d**) Venn diagram describing the overlap in CNVs that have been confirmed by CMA and at the same time detected by three tools. An overlapping CNV was defined as at least one exon that shared at least 50% of its overall length within a CNV region called by different tools

Among the 171 variants detected by CMA, 152 variants were detected by at least one WES program. Forty-six variants were detected by XHMM and CoNIFER, which are shown in Fig. 3(A). We plotted the size ratio of those detected from three programs, using CMA as reference. XHMM and CoNIFER detected more accurate size of variants, while CNVnator reported a significantly larger CNV size (Fig. 3(B)).

4. Characteristics of CNV missed by exome data (Fig. 3 (C))

A large number of CNVs (125) were missed by CoNIFER and XHMM combined detection and were further investigated. The WES read coverage and capture probes distribution were insufficient for both CoNIFER and XHMM detection of 42 CNVs. XHMM and CoNIFER automatically filtered capturing probes located in the region within recurrent CNV detected in the same batch; thus, 43 variants were missed and had to be confirmed with involving probes. Details of all 171 variants are available in the supplemental data (Additional file 2: Table S2).

5. Poor concordance among three programs (Fig. 3 (D))

Although CoNIFER and XHMM used a similar batched input approach, poor CNV detection concordance was still identified in our study. CNVnator discovered most variants and covered most variants that could be detected through WES data. Only 17 CNVs were detected by all three programs.

6. Detection of clinical relevant variants

All variants were evaluated with our in-house standard for clinical relevant variants and eight CNVs were categorized as pathogenic or likely pathogenic variants ranging from 306 kb – 35 mb. Six of these variants were detected by WES programs. A 306 kb variant on chromosome 3 remained undetected due to particularly low capture probe coverage within the variant region. Another 11 mb variant on chromosome 2 remained undetected despite sufficient capture probes and depth coverage (Additional file 3: Table S3).

## Discussion

Copy number variants (CNVs) are a very important target in the clinical diagnosis of genetic diseases. CMA has been proven as the most stable and accurate platform for CNV detection and has been implemented as a clinical test for more than a decade. NGS now provided a new approach for detecting CNV, which can potentially replace CMA. Before implementing NGS-based CNV detection, extensive validation is required to evaluate the validity of the new method.

Numerous WES based CNV detection programs have been developed, including the 15 read-depth based CNV detection tools currently available [21]. We selected three representative and well-known methods for this study. XHMM is the most commonly accepted software, which employs the classical hidden Markov model (HMM) for CNV identification and achieves a sensitivity of 8–14% via XHMM, reported against CNV detection based on WGS data [13]. The XHMM framework starts with aligned BAM files to calculate the depth of coverage; then, utilizing normalized read depths via principal component analysis (PCA). Finally, XHMM uses the normalized data to train and run a Hidden Markov Model (HMM) for CNV detection. CoNIFER was the first developed tool to deal with rare CNVs from multiple samples and has been chosen as representative software, which can be used as reference in evaluating other new softwares [22]. CoNIFER calculates the RPKM (reads per kilobase per million mapped reads) values for each sample, and utilizes the singular value decomposition (SVD) method (originating from linear algebra) to reduce data dimensions for detecting obvious CNV signals. Evaluation against the array CGH platform in breast cancer samples characterized CoNIFER as leading to high false positives, low sensitivity, and obvious duplication bias [11]. Another study showed that CoNIFER achieves higher precision, but at a cost of reduced sensitivity below 5% [13]. XHMM and CoNIFER have been evaluated in parallel in patients with nonsyndromic hearing loss showing poor concordance on size of detected CNV [23]. However, both tools are noted for advantages of identification of rare CNV from a population of WES samples [24]. CNVnator was previously used in whole genome data for CNVs identification based on read depth, and was accessed to achieve better resolution of CNV borders than the other WGS data-based tools [25]. The main methodology for CNVnator is a mean-shift. The software first divides the whole genome into equal sized, non-overlapping bins, and treats the mapped reads of each bin as a read depth signal. To estimate copy number change in each genome segment, it then calculates the *P*-value for a one-sample t-test, testing whether the mean RD signal of a segment would be close to the genome average. In a comprehensive comparison study, CNVnator was accessed to be outstanding in break point position and copy number estimation; however, disconcordance of variants was also discovered among all tools evaluated in the study [26].

In our study, large differences were observed in number and size distribution of CNVs detected from CMA and three WES based tools. Microarray platforms have a smaller capacity to detect small variants that are not covered by a sufficient number of probes. Several studies have tried to understand the roles of these small variants. The detection of small, non-recurrent pathogenic or likely pathogenic CNVs could help to increase the diagnostic yield of CMA clinical testing by ~3% [27, 28]. WES-based tools, such as XHMM and CoNIFER, are capable of detecting small variants as long as a sufficient number of capturing probes (> 10) are covered in the region and enable a sensitivity of 14.6 and 22.2%, respectively, indicating the importance of probe number for CNVs detection. The overwhelming number of variants CNVnator detected from samples was due to the extreme resolution of the algorithm [19]. This extreme resolution is affected by sequencing depth and high resolution could result in splitting large CNVs into small pieces, which are more sensitive in detecting smaller variants. Larger bin size setting in CNVnator could help to merge consecutive small CNVs as integrated variants; however, this parameter was limited by the average sequencing depth of our clinical WES data.

125 CMA confirmed CNVs that were not detected by XHMM and CoNIFER were further investigated for possible explanations. Low sequencing depth (< 10 X) and limited capture probes (< 10) were detected in 42 variants and these regions were automatically excluded during the normalization step of both tools. The detection for these CNV may be improved if sequence depth increased. The programs filter out capture probes located in recurrent variants that detected the same batch during data processing; thus, 43 polymorphism CNVs were neglected during the detection, which was also confirmed by our in-house array database [http://database.gdg-fudan.org/DB_HTML/DataSub.html]. Thus, only 40 (23.4%) CNVs remained theoretically undetected. Limitation of sample number and sequencing depth of XHMM and CoNIFER could be a possible explanation of these undetected variants. CoNIFER requires at least 50 million mapped reads and a minimum of eight exome samples to run at a time, while XHMM recommends ~50 exome samples with at least 60–100 X coverage [18, 29]. Characteristics of samples in each batch also contribute to the effectiveness of CNV detection in XHMM and CoNIFER. Recurrent pathogenic or likely pathogenic variants may be filtered out erroneously, if they existed in multiple samples; therefore, including non-abnormal reference samples as part of the batch could help to detect these CNVs. Conservative predefined thresholds in default settings of the CoNIFER might be a further reason for missing variants. Read-depth based tools are fairly limited to repeated regions of the reference genome [30]; thus, the sequence nature of specific locations also hinders detection of variants. CNVnator was designed for CNV discovery and genotyping from read-depth analysis based on a mean-shift approach. The number of nucleotides covered in each shift is called bin size (50–60 in our study), which can be determined by the average coverage of sequencing data (45–70 for our samples). CNVnator had the highest sensitivity of 87.7% since 150 of 171 CMA confirmed variants were detected by CNVnator. A Venn diagram was used to show the poor disconcordance among WES based tools, which was attributed to unsatisfying sequencing depth and inadequate number of batched samples. Therefore, CNV detection from WES based tools was affected by the following factors (ranked in the order of importance): probe number, reading depth, sample constituent in the batch, software parameters, and sequence nature of variants.

Using CMA detected variants as standard, the three tested WES based CNV detecting tools were not able to detect the accurate size of variants from WES data. XHMM and CoNIFER have lower sensitivity, but more accurate size of CNVs compared to CNVnator. CNVnator reached higher sensitivity at the cost of high false positive rates and exaggerated readout of the variant size. Poor concordance of CNV detection was observed in the study. Increasing the number of batch samples and valid sequencing depth were the most realizable approaches to improve performance of these WES based tools. At this stage, CMA still remains the first-choice and gold standard for CNV detection for clinical diagnostic purpose. CNV detection tools using WES data could be used as a screening tool.

## Conclusion

Low concordances of CNV detection were observed via three different read-depth based programs indicating that WES-based CNV detection still remains immature and unstable compared to CMA. Since WES based CNV detection was evaluated to have low sensitivity and uncertain specificity in comparison with CMA based CNV detection, CMA will continue to play an important role in detecting clinical grade CNV in the NGS era, which is largely based on WES. CNV detection tools using WES data could be considered as a complementary way with only computational effort, but where further validation has been suggested for the purpose of clinical diagnosis.

## Additional files

Additional file 1: Details and quality control information of sequen﻿cing data. (XLS 32 kb)

Additional file 2: Details of all 17﻿1 variants detected by CMA. (XLS 90 kb)

Additional file 3: Details of eight clinical relevant CNVs. (XLS 37 kb)
